# The form of density-dependence in host growth impacts parasitoid-driven host suppression

**DOI:** 10.1371/journal.pone.0358117

**Published:** 2026-11-05

**Authors:** Abhyudai Singh, Poulami Chatterjee

**Affiliations:** 1 Department of Electrical and Computer Engineering, University of Delaware, Newark, Delaware, United States of America; 2 Department of Biomedical Engineering, University of Delaware, Newark, Delaware, United States of America; 3 Department of Mathematical Sciences, University of Delaware, Newark, Delaware, United States of America; 4 Center for Bioinformatics and Computational Biology, University of Delaware, Newark, Delaware, United States of America; National Museums of Kenya, KENYA

## Abstract

Leveraging the discrete-time formalism of the Nicholson-Bailey model, we investigate the ecological population dynamics of host-parasitoid interactions with an arbitrary form of density-dependence in the host growth rate, and map out regimes allowing stable coexistence of the consumer (parasitoid) and the resource (host). If the parasitoid-free host population dynamics is stable, we provide sufficient conditions that ensure stability is maintained with the inclusion of the parasitoid irrespective of the level of parasitism. When parasitoids parasitize hosts at a constant rate, high parasitism levels are shown to always destabilize the population dynamics. This results in a limit of host suppression that we quantify using the ratio of host density (just before stability is lost for high parasitism levels) and the host’s parasitoid-free carrying capacity. We systematically examine this limit for different types of intrinsic self-limitations in host growth. Our analysis shows that for both Ricker and Beverton-Holt-type host population dynamics, this parasitoid-driven host suppression limit is 33% for slow-growing hosts and 10−20% for fast-growing hosts. Moreover, our results reveal that these limits can be much higher for Hill-type density-dependence in host growth rate. In summary, this contribution characterizes the impact of coupling different forms of intrinsic host population dynamics with and without additional stabilizing factors (such as a fraction of hosts protected from parasitism). These results have important implications for using parasitoids as natural enemies against arthropod pest populations, and the success of such biological control is inherently tied to the form of self-limitation in host growth.

## Introduction

Parasitoid wasps are obligate parasites that ultimately kill their arthropod hosts and represent an ecologically significant group of natural enemies in pest management programs worldwide [1–5]. Since the seminal work of Nicholson and Bailey close to a century ago [6], mathematical models have provided rich insights into ecological mechanisms that stabilize or destabilize host-parasitoid population dynamics [5]. Here we use models to address an important question in the context of biological control: are there limits to parasitoid-driven host (pest) suppression that destabilize population dynamics? We address this question for general forms of self-limitation in host growth that set the parasitoid-free host carrying capacity. Furthermore, we explore this limit as a function of ecologically relevant parameters, such as host reproduction and parasitoid handling times. We next review the mathematical formalism used to capture host-parasitoid interactions.

The classical framework for modeling host-parasitoid population dynamics is the discrete-time formalism of the Nicholson-Bailey model [5–11] that takes the form


$$\begin{array}{cc} H_{t+1} & =RH_{t}e^{-cTP_{t}}, \end{array}$$ (1a)

$$\begin{array}{cc} P_{t+1} & =kRH_{t}\left(1-e^{-cTP_{t}}\right). \end{array}$$ (1b)

In this model, $H_{t}$ and $P_{t}$ are the population densities of the adult host and parasitoid, respectively, in year t∈{1,2,3,...}. *R* > 1 quantifies the host reproduction and the absence of any host growth self-limitation in (1) results in geometric growth in host density


$$\begin{array}{c} H_{t+1}=RH_{t}\;⟹\;H_{t}\propto R^{t}, \end{array}$$ (2)

when the parasitoid is not present ($P_{t}=0$). Key assumptions of the Nicholson-Bailey model are that parasitoids are not egg-limited and search for hosts randomly, parasitizing them at a constant rate *c* with fast handling times corresponding to a Type I functional response. We refer to *c* as the *parasitoid attack rate*, and 1/*c* can be interpreted as the average time taken by a parasitoid to search, locate, and successfully parasitize a host. In (1), *T* denotes the overall period of host vulnerability to parasitoid attacks, and without loss of generality, we will set it as *T* = 1. Finally, k≥1 is the number of adult hosts emerging from a single parasitized host.

Apart from the trivial fixed point $\left(H_{t},P_{t})=(0,0\right)$ which is unstable for *R* > 1, the model (1) has a unique non-trivial fixed point


$$\begin{array}{c} H^{*}=\frac{\log(R)}{(R-1)kc},\;P^{*}=\frac{\log(R)}{c} \end{array}$$ (3)

where both species are present, and log represents the natural logarithm. It is well known that this fixed point is unstable, resulting in population density oscillations that increase in amplitude over time, eventually leading to parasitoid extinction [12]. A myriad of ecological mechanisms have been characterized that stabilize the host-parasitoid population dynamics including, some hosts being protected from parasitism [13], host-to-host differences in susceptibility to parasitism [14–20], and host feeding [21]. While Type III functional responses have a stabilizing effect under specific parameter regimes [22–24], Type II responses are destabilizing [5,25].

In this contribution, we investigate the role of density-dependent host growth that sets the host density at a carrying capacity in the absence of the parasitoid. Such self-limitation in host growth has been known to stabilize the host-parasitoid interaction [26–31]. Interestingly, the relative timing of parasitism with respect to when the self-limitation acts on the host population critically influences the population dynamics [32]. Recent works have used “semi-discrete” frameworks leveraging continuous-time differential equation models of host parasitism to obtain yearly update functions in discrete-time models to explore host self-limitation acting concurrently with parasitoid attacks [24,33]. Moreover, a systematic comparison of different scenarios reveals that parasitism acting after host-growth limitation provides the most efficient stable suppression of host population density below the carrying capacity [34]. Such a scenario can arise when, for example, self-limitation in host growth arises from resource competition at the larval stage, and parasitoids attack the host pupal stage (Fig 1).

**Fig 1:**
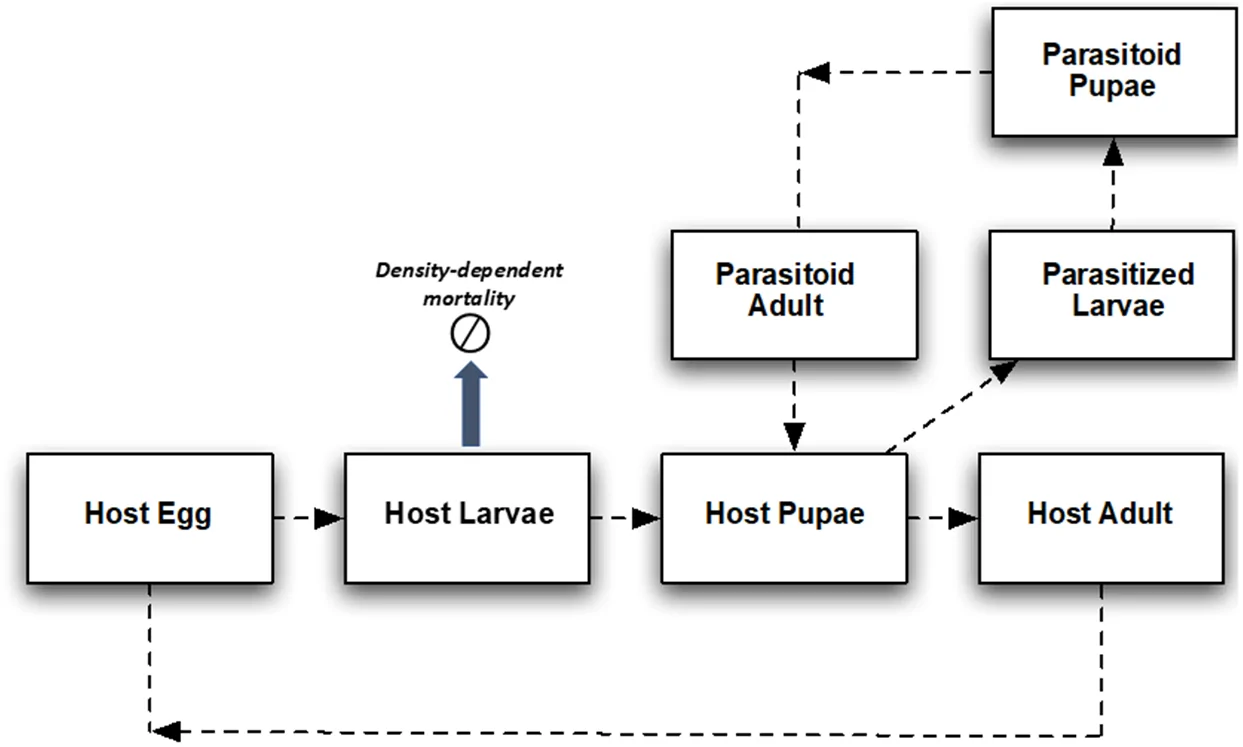
A schematic of the host-parasitoid life cycle corresponding to the model (18), where self-limitation in host growth occurs at a stage before parasitoid attack. While the model structure is quite general, we here show an illustrative ecological example of this model where self-limitation in host growth occurs at the larval stage due to density-dependent mortality as a result of either predation or resource competition. Parasitoids attack hosts at the pupal stage after host self-limitation has acted on the population.

Considering an arbitrary form of self-limitation in host growth, we mathematically characterize the region allowing stable coexistence of both the host and the parasitoid. When parasitoids attack hosts at a constant rate, sufficiently large parasitism levels always destabilize the population dynamics and we explore the limit to which host density can be suppressed while still ensuring a stable consumer-resource coexistence. Intriguingly, our analysis shows that for a general class of models describing the host’s population dynamics, this limit is always one-third of the host’s parasitoid-free population density when R≈1, and the limit further decreases with increasing values of *R*. Our results also characterize stability regions when density-dependent host growth is combined with density-dependent parasitoid search efficiency ensuring a stable population interaction even for high levels of parasitism that suppress host density to arbitrarily low levels.

## Modeling background

### Host population dynamics

In the parasitoid’s absence, the population dynamics of the host species follow the discrete-time model


$$\begin{array}{c} H_{t+1}=RH_{t}f_{H}(H_{t}),\;R>1 \end{array}$$ (4)

where the self-limitation in host growth is captured via the function $f_{H}(x)\in [0,1]$ that is defined for x≥0. This self-limitation could arise as a result of either intraspecific competition for resources or density-dependent predation by consumers other than the specific parasitoid in consideration. $f_{H}$ is assumed to be a *continuously differentiable* and a *monotonically decreasing* function with $f_{H}(0)=1$. An ecologically relevant form for this function is


$$\begin{array}{c} f_{H}(H_{t})=\frac{1}{{\left(1+\frac{\left(R^{\frac{1}{q}}-1\right)H_{t}}{K}\right)}^{q}} \end{array}$$ (5)

where *K*,*q* > 0, and specific values of the quotient *q* correspond to different classical models of single-species population dynamics [35–37]. For example, *q* = 1 results in the Beverton-Holt model


$$\begin{array}{c} H_{t+1}=\frac{RH_{t}}{1+\frac{\left(R-1\right)H_{t}}{K}}. \end{array}$$ (6)

Using the fact that


$$\begin{array}{c} \lim_{q\to \infty }{\left(1+\frac{\left(R^{\frac{1}{q}}-1\right)H_{t}}{K}\right)}^{q}=R^{\frac{H_{t}}{K}}, \end{array}$$ (7)

in the limit q→∞, (4)-(5) converge to the Ricker model [38]


$$\begin{array}{c} H_{t+1}=H_{t}R^{1-H_{t}/K}. \end{array}$$ (8)

Given the convergence of (5) to classical host population dynamics models for *q* = 1 and q→∞, values of *q* > 1 can be used to interpolate between the Beverton-Holt and Ricker models. Note that the slope of the function $f_{H}(x)$ at *x* = 0 for $f_{H}$ given by (5) is


$$\begin{array}{c} \frac{df_{H}(x)}{dx}{|}_{x=0}=-\frac{q\left(R^{\frac{1}{q}}-1\right)}{K}, \end{array}$$ (9)

and this slope becomes increasingly negative as *q* decreases. Thus, values of 0 < *q* < 1 would correspond to functions $f_{H}$ that decline rapidly with increasing host density at lower densities but show a shallower decline at higher host densities compared to the Beverton-Holt model (see *q* = 0.2 in Fig 2).

**Fig 2:**
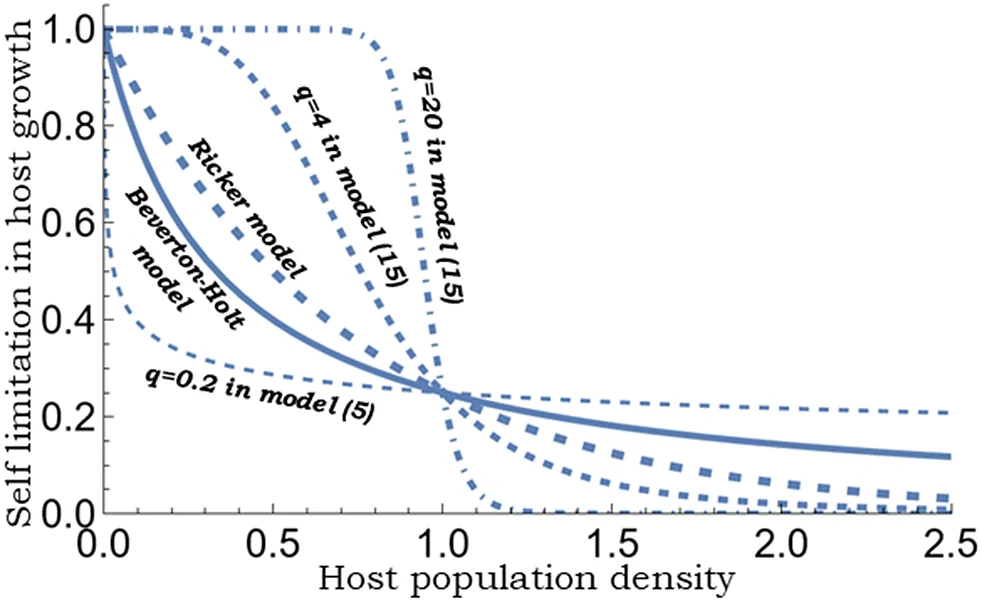
Comparison of different functions $f_{H}(H_{t})$ used in model (4) to describe self-limitation in host growth. The plot compares the Beverton–Holt model (*q* = 1 in (5)), the Ricker model (q→∞ in (5)), model (5) with *q* = 0.2, and model (15) with *q* = 4 and *q* = 20. Increasing *q* in (15) changes the shape of the density-dependent response, with the decline in $f_{H}$ occurring at higher host densities.

The model (4) is assumed to have a unique non-trivial fixed point $H_{t}=K$ that represents the host’s parasitoid-free population density satisfying


$$\begin{array}{c} f_{H}(K)=\frac{1}{R}. \end{array}$$ (10)

It is convenient to characterize the stability of this fixed point in terms of the dimensionless quantity


$$\begin{array}{c} S_{H}(x):=\frac{x}{f_{H}(x)}\frac{df_{H}(x)}{dx}, \end{array}$$ (11)

that is the log sensitivity of the function $f_{H}$ with respect to the host population density and can be interpreted as the strength of density-dependent host growth. The fixed point $H_{t}=K$ is asymptotically stable, if and only if,


$$\begin{array}{c} -2<S_{H}(K)<0 \end{array}$$ (12)

[39]. For $f_{H}$ as given by (5), the log sensitivity takes the form


$$\begin{array}{c} S_{H}(H_{t})=-q\left(1-\frac{1}{1+\frac{\left(R^{\frac{1}{q}}-1\right)H_{t}}{K}}\right)\;⟹\;S_{H}(K)=-q\left(1-\frac{1}{R^{\frac{1}{q}}}\right). \end{array}$$ (13)

Given that *R* > 1, one can see that the stability condition (12) is always satisfied for 0<q≤2. Thus, the fixed point $H_{t}=K$ is stable for any level of host reproduction when 0<q≤2. When *q* > 2, (12) is satisfied for


$$\begin{array}{c} 1<R<{\left(\frac{q}{q-2}\right)}^{q} \end{array}$$ (14)

with the range of *R* allowing stability shrinking with increasing *q*. For example, when *q* = 3, (14) corresponds to 1 < *R* < 27, and when q→∞ (the Ricker model), it shrinks to $1<R<e^{2}\approx 7.39$.

Another model formulation of host self-limitation that we will use in the manuscript is


$$\begin{array}{c} f_{H}(H_{t})=\frac{1}{1+(R-1){\left(\frac{H_{t}}{K}\right)}^{q}}\;⟹\;H_{t+1}=\frac{RH_{t}}{1+(R-1){\left(\frac{H_{t}}{K}\right)}^{q}}, \end{array}$$ (15)

which considers a Hill equation formulation of $f_{H}$ with *q* corresponding to the Hill coefficient. While both (5) and (15) are identical for *q* = 1 (Beverton-Holt model), a difference arises when q≫1. In (15), large values of *q* result in a nonlinear function $f_{H}$ that remains close to one when host density is below a threshold but sharply decreases to zero when host density crosses the threshold (Fig 2). Such intrinsic self-limitation in host growth can be shaped by mechanisms such as frequency-dependent predation and prey switching, where a predator disproportionately consumes the host as it becomes more abundant [40–43]. Evaluating the dimensionless log-sensitivity of (15) yields,


$$\begin{array}{c} S_{H}(H_{t})=-q\left(1-\frac{1}{1+(R-1){\left(\frac{H_{t}}{K}\right)}^{q}}\right)\;⟹\;S_{H}(K)=-q\left(1-\frac{1}{R}\right). \end{array}$$ (16)

In this case, using (12), the fixed point $H_{t}=K$ is always stable for 0<q≤2, and stable for


$$\begin{array}{c} 1<R<\frac{q}{q-2} \end{array}$$ (17)

when *q* > 2.

### Host-parasitoid population dynamics

Having defined the host population dynamics, we now introduce the parasitoid, resulting in the two-dimensional consumer-resource model


$$\begin{array}{cc} H_{t+1} & =RH_{t}f_{H}(H_{t})f_{P}(P_{t}) \end{array}$$ (18a)

$$\begin{array}{cc} P_{t+1} & =kRH_{t}f_{H}(H_{t})\left(1-f_{P}(P_{t})\right), \end{array}$$ (18b)

where the function $f_{P}(P_{t})$ is the *escape response* quantifying the fraction of hosts escaping parasitism to become adult hosts for the following year. The model makes several key assumptions:

- The escape response $f_{P}(P_{t})$ is only a function of the parasitoid density and hence excludes Type II and III functional responses.
- The function $f_{P}(P_{t})$ is a *continuously differentiable* and a *monotonically decreasing* function with $f_{P}(0)=1$. Thus, the fraction of hosts escaping parasitism decreases with increasing parasitoid density $P_{t}$.
- Parasitism occurs after hosts have been subjected to intrinsic growth limitation (Fig 1). In this case, $RH_{t}f_{H}(H_{t})$ is the host density vulnerable to parasitism; $f_{P}$ and $1-f_{P}$ are the unparasitized and parasitized host fractions, respectively, at the end of the vulnerable period.

The consumer-resource model (18) has a fixed point $\left(H_{t},P_{t})=(K,0\right)$ that excludes the parasitoid. However, we are primarily interested in another fixed point $\left(H_{t},P_{t})=(H^{*},P^{*}\right)$ where $H^{*}>0$ and $P^{*}>0$ allowing coexistence of both species. Setting $H_{t+1}=H_{t}=H^{*}$ and $P_{t+1}=P_{t}=P^{*}$ in (18) shows that this fixed point is the solution to


$$\begin{array}{c} \frac{1}{R}=f_{H}(H^{*})f_{P}(P^{*}),\;H^{*}=\frac{P^{*}f_{P}(P^{*})}{k\left(1-f_{P}(P^{*})\right)}. \end{array}$$ (19)

To assess the stability of a fixed point, we first obtain the corresponding Jacobian matrix *A* by linearizing the nonlinearities on the right-hand side of (18) around the given fixed point. For the fixed point $\left(H_{t},P_{t})=(K,0\right)$ corresponding to parasitoid extinction and the host density at the carrying capacity, *K*, the detailed Jacobian calculation is mentioned in S1 Appendix A in S1 File. This analysis shows that the fixed point $\left(H_{t},P_{t})=(K,0\right)$ is stable if and only if


$$\begin{array}{c} -2<S_{H}(K)<0,\;-1<kKf_{P}^{\prime }(0leq0. \end{array}$$ (20)

Recall that the first inequality in (20) is the same as in (12), while the second inequality arises from the fact that when $H_{t}=K$,


$$\begin{array}{c} \lim_{P_{t}\to 0}\frac{P_{t+1}}{P_{t}}=-kKf_{P}^{\prime }(0). \end{array}$$ (21)

and $-kKf_{P}^{\prime }(0)<1$ leads to parasitoid extinction starting from small parasitoid densities. This result leads to a condition for parasitoid establishment:


$$\begin{array}{c} -kKf_{P}^{\prime }(0)>1, \end{array}$$ (22)

is a necessary and sufficient condition for parasitoids to grow from small numbers.

Having established the condition under which parasitoids can invade from low densities, we next examine the positive equilibrium at which both host and parasitoid populations persist. The detailed calculation to find the Jacobian and stability criteria is shown in S1 Appendix B in S1 File. A stable host-parasitoid coexistence occurs if and only if


$$\begin{array}{cc} & 1-f_{P}(P^{*})+S_{P}(P^{*})>-S_{H}(H^{*})S_{P}(P^{*}), \end{array}$$ (23a)

$$\begin{array}{cc} & S_{H}(H^{*})(-1+f_{P}(P^{*})-S_{P}(P^{*}))-\left(1-f_{P}(P^{*})\right)S_{P}(P^{*})>0, \end{array}$$ (23b)

$$\begin{array}{cc} & S_{H}(H^{*})(1-f_{P}(P^{*})-S_{P}(P^{*}))+2\left(1-f_{P}(P^{*})\right)-S_{P}(P^{*})\left(1+f_{P}(P^{*})\right)>0, \end{array}$$ (23c)

where $S_{H}$ and $S_{P}$ are the dimensionless log sensitivities of the functions $f_{H}$ and $f_{P}$, respectively,


$$\begin{array}{c} S_{H}(H^{*}):=\frac{H_{t}}{f_{H}(H_{t})}\frac{df_{H}(H_{t})}{dH_{t}}{|}_{H_{t}=H^{*}}<0,\;S_{P}(P^{*}):=\frac{P_{t}}{f_{P}(P_{t})}\frac{df_{P}(P_{t})}{dP_{t}}{|}_{P_{t}=P^{*}}<0. \end{array}$$ (24)

We investigate these inequalities in further detail below for different scenarios, highlighting new results and connecting them with known results.

#### Absence of self-limitation in host growth.

To review classical stabilizing mechanisms of host-parasitoid population dynamics, we briefly consider the scenario of no intrinsic self-limitation in host growth. In the absence of any intrinsic self-limitation in host growth


$$\begin{array}{c} f_{H}(H_{t})=1⟹S_{H}(H^{*})=0, \end{array}$$ (25)

from (19) the coexistence fixed point is given as the unique solution to


$$\begin{array}{c} \frac{1}{R}=f_{P}(P^{*}),\;H^{*}=\frac{P^{*}}{k(R-1)}. \end{array}$$ (26)

Using $S_{H}(H^{*})=0$, and given that $f_{P}(P^{*})<1$ and $S_{P}(P^{*})<0$, the last two inequalities in (23) are always true, and stability is tied to the first inequality (23a). In this case, the coexistence fixed point is stable if and only if


$$\begin{array}{c} 1-f_{P}(P^{*})+S_{P}(P^{*})>0. \end{array}$$ (27)

For the Nicholson-Bailey model,


$$\begin{array}{c} f_{P}(P^{*})=e^{-cP^{*}},\;S_{P}(P^{*})=-cP^{*}, \end{array}$$ (28)

inequality (27) is always violated for any $P^{*}>0$ and, hence, the unstable Nicholson-Bailey fixed point. We next review three complementary scenarios that drive stability and relate them to (27).

***Parasitoid interference***: Interference between parasitoids can be captured using a density-dependent attack rate,


$$\begin{array}{c} c=c_{m}P_{t}^{-m},\;c_{m}>0,\;0<m<1, \end{array}$$ (29)

and this power-law-based decrease in search efficiency with parasitoid density has been empirically observed for many parasitoids [44]. This leads to


$$\begin{array}{c} f(P_{t})=e^{-c_{m}P_{t}^{1-m}}⟹\;S_{P}(P_{t})=-(1-m)c_{m}P_{t}^{1-m}. \end{array}$$ (30)

Using the fact that at equilibrium $f(P^{*})=e^{-c_{m}{\left(P^{*}\right)}^{1-m}}=1/R$, inequality (27) simplifies to


$$\begin{array}{c} 1-f_{P}(P^{*})+S_{P}(P^{*})>0\;⟹\;1-\frac{1}{R}-(1-m)c_{m}{\left(P^{*}\right)}^{1-m}>0\;⟹\;1-\frac{1}{R}-(1-m)\log R>0, \end{array}$$ (31)

resulting in a stable species coexistence when


$$\begin{array}{c} 1-\frac{R-1}{R\log R}<m<1. \end{array}$$ (32)

For example, when *R* = 2 this range is 0.28 < *m* < 1, and shrinks to 0.68 < *m* < 1 for *R* = 20.

***Fractional host refuge***: One known mechanism that stabilizes the Nicholson-Bailey fixed point is when a given fraction μ of hosts are protected from parasitism [13]. In this case, the functional response and its log sensitivity take the form


$$\begin{array}{c} f(P_{t})=\mu +(1-\mu )e^{-cP_{t}}\;⟹\;S_{P}(P_{t})=-\frac{(1-\mu )cP_{t}e^{-cP_{t}}}{\mu +(1-\mu )e^{-cP_{t}}}, \end{array}$$ (33)

respectively. When $f_{H}(H_{t})=1$ and μ<1/R, the resulting model has a unique non-trivial fixed point given by


$$\begin{array}{c} H^{*}=\frac{\log\left(\frac{(1-\mu )R}{\left(1-\mu R\right)}\right)}{(R-1)kc},\;P^{*}=\frac{\log\left(\frac{(1-\mu )R}{\left(1-\mu R\right)}\right)}{c}, \end{array}$$ (34)

that is asymptotically stable for a range of refuge fractions


$$\begin{array}{c} \mu^{*}<\mu <1/R, \end{array}$$ (35)

where $\mu^{*}$ is the unique solution of


$$\begin{array}{c} \frac{R-1}{R(1-\mu^{*}R)}=\log\left(\frac{R(1-\mu^{*})}{1-\mu^{*}R}\right) \end{array}$$ (36)

[5]. As recently shown in [34], when R≈1, the range (35) can be approximated by


$$\begin{array}{c} \frac{1}{2R}<\mu <\frac{1}{R}. \end{array}$$ (37)

***Variation in host risk***: The concept of host refuge can be generalized by considering an arbitrary distribution of parasitism risk across the host population [14–19]. Assuming the attack rate *c* in the Nicholson-Bailey model to be a random variable with a probability distribution function (pdf) *p*(*x*) results in the escape response


$$\begin{array}{c} f(P_{t})=\int_{x=0}^{\infty }p(x)\exp(-xP_{t}). \end{array}$$ (38)

Considering *p*(*x*) to be a Gamma distribution with mean *c* and coefficient of variation *CV* (standard deviation divided by the mean) transforms (38) to


$$\begin{array}{c} f(P_{t})=\frac{1}{{\left(1+cCV^{2}P_{t}\right)}^{1/CV^{2}}}⟹\;S_{P}(P^{*})=-\frac{cP^{*}}{1+CV^{2}cP^{*}}. \end{array}$$ (39)

Note that when *CV* = 1


$$\begin{array}{c} 1-f_{P}(P^{*})+S_{P}(P^{*})=0 \end{array}$$ (40)

and inequality (27) is satisfied for any $P^{*}>0$, if and only if, *CV* > 1. Thus, sufficient variability in host susceptibility to parasitism that leads to aggregation of parasitoid attacks to high-risk hosts leads to stable population dynamics [45–47]. Further works have shown that when R≈1, then *CV* > 1 is necessary and sufficient for stability independent of the form of *p*(*x*). However, for larger values of *R*, the shape of the risk distribution, *p*(*x*), must have zero modal risk for stability [48].

#### The Beverton-Holt model.

As can be seen from (13), for the Beverton-Holt model where *q* = 1,


$$\begin{array}{c} -1<S_{H}(H^{*})<0, \end{array}$$ (41)

in which case a necessary and sufficient condition for stable coexistence is


$$\begin{array}{c} 1-f_{P}(P^{*})+S_{P}(P^{*})>-S_{H}(H^{*})S_{P}(P^{*}) \end{array}$$ (42)

as the last two inequalities in (23) are always satisfied. Since both the log sensitivities are negative,


$$\begin{array}{c} 1-f_{P}(P^{*})+S_{P}(P^{*})>0 \end{array}$$ (43)

is a sufficient condition for stable coexistence.

## Results

### Limits of host density suppression for constant parasitoid attack rate

In this section, we investigate model (18) with an arbitrary form of density-dependence in host growth and escape response $f(P_{t})=e^{-cP_{t}}$ as in the Nicholson-Bailey model. Recall that parasitoid establishment (the ability of the consumer to grow from small densities (22)) requires


$$\begin{array}{c} -kK\frac{df_{P}(P_{t})}{dP_{t}}{|}_{P_{t}=0}>1\;⟹\;c>\frac{1}{kK}. \end{array}$$ (44)

Using (28), one can map the stability region as determined by the inequalities (23) as a function of $S_{H}$ and $cP^{*}$ (Fig 3).

**Fig 3:**
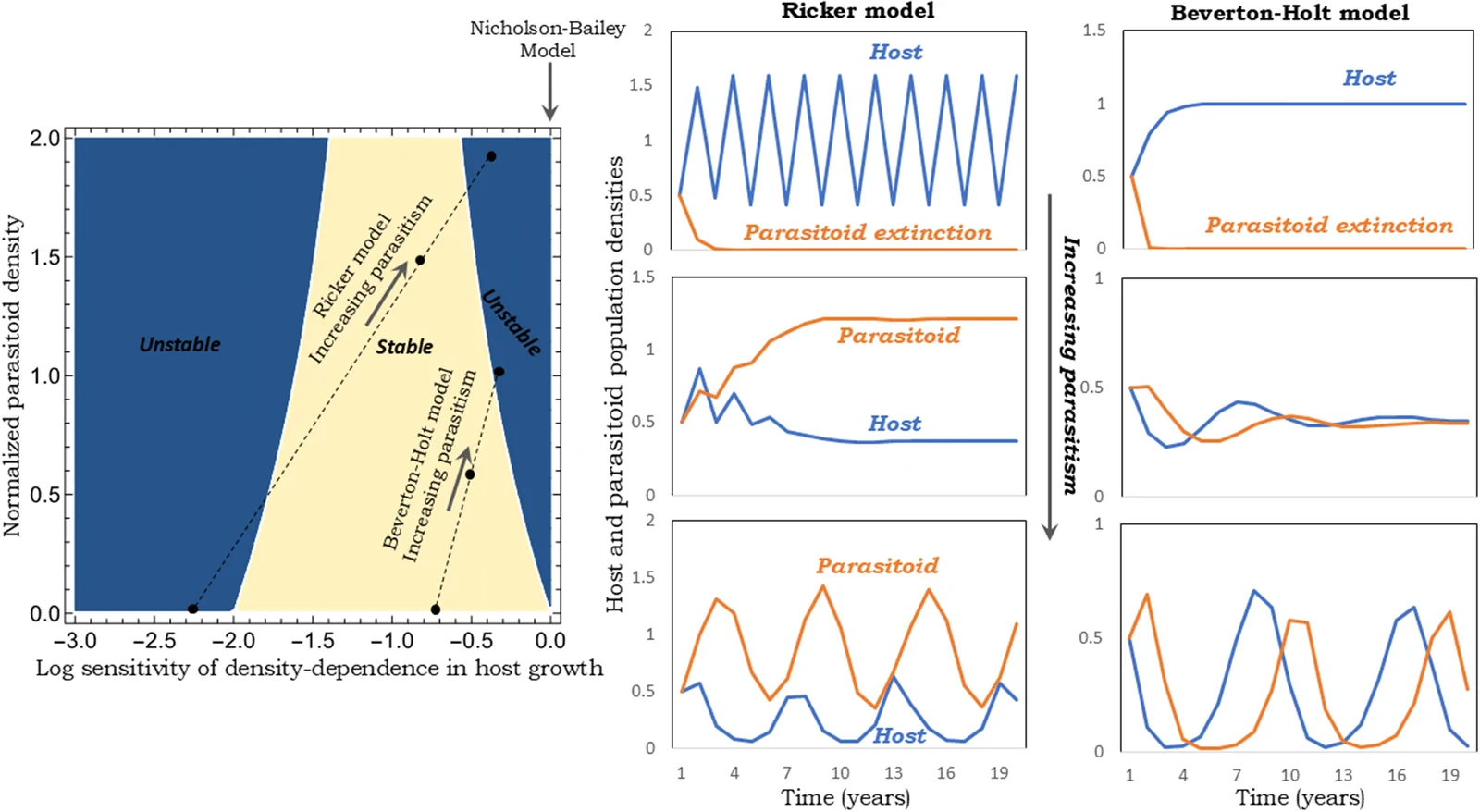
Region of stable host-parasitoid coexistence for the model (18) as given by (23) for the Nicholson-Bailey escape response $f(P_{t})=e^{-cP_{t}}$. Here, the stability region is mapped in terms of $S_{H}(H^{*})$ (the strength of density-dependence in host growth) on the x-axis and the parasitoid level $cP^{*}$ on the y-axis. Stability arises for a range of values of $S_{H}(H^{*})$ with the instability-stability boundaries on the right and left defined by inequalities (23a) and (23c), respectively. The figure shows the paths traced by the Beverton-Holt and Ricker models of intrinsic host population dynamics as parasitoid attack rates increase, reducing host density and ultimately destabilizing the system dynamics. Parameters are taken as *k* = *K* = 1, *R* = 4 for the Beverton-Holt model, and *R* = 9.5 for the Ricker model. Population trajectories corresponding to the black circles on the stability region are shown on the right for increasing parasitoid attack rates. Note for the Ricker model the host population dynamics is unstable in the parasitoid’s absence. The inclusion of the parasitoid stabilizes the population dynamics, which is again destabilized for a sufficiently large attack rate.

In Fig 3, the x-axis $S_{H}=0$ corresponds to the unstable Nicholson-Bailey model. The stabilizing effect of self-limitation in host growth is clearly seen in the figure, where making $S_{H}$ negative leads to stable population dynamics, with further negative values again destabilizing the system. The range of stabilizing values of $S_{H}$ shrinks with increasing levels of parasitoids (Fig 3). Here the instability-stability boundary on the right and left arise from inequalities (23a) and (23c), respectively, and these boundaries converge to $S_{H}=-1$ in the limit $cP^{*}\to \infty$.

Note that in this case, a sufficiently large parasitoid attack rate will drive the host density much below the carrying capacity with $f_{H}\approx 1$, reducing the model to the unstable Nicholson-Bailey model. This point is illustrated in Fig 3 for both Beverton-Holt and Ricker models of self-limitation in host growth, where sufficiently large attack rates destabilize the system by crossing the boundary defined by the inequality (23a). This sets up an interesting question: what is the fundamental limit to which the host density can be suppressed below its parasitoid-free density and how does this depend on the form of $f_{H}$?

Mathematically, this limit can be determined from the stability-instability boundary as given by (23a)


$$\begin{array}{c} 1-f_{P}(P^{*})+S_{P}(P^{*})=-S_{H}(H^{*})S_{P}(P^{*}) \end{array}$$ (45)

where at equilibrium


$$\begin{array}{c} f_{P}(P^{*})=e^{-cP^{*}}=\frac{1}{Rf_{H}(H^{*})}⟹cP^{*}=\log Rf_{H}(H^{*}) \end{array}$$ (46)

and


$$\begin{array}{c} S_{P}(P^{*})=-cP^{*}=-\log Rf_{H}(H^{*}). \end{array}$$ (47)

For a given functional form $f_{H}$, the minimal value of $H^{*}$ conditioned on stable host-parasitoid coexistence can be obtained by substituting (46)-(47) in (45) and solving for $H^{*}$. Quantifying the limit of host suppression as the ratio $H^{*}/K$ – the minimal achievable stable host density normalized to its parasitoid-free density, our analysis shows that the limit decreases with increasing *R* (Fig 4).

**Fig 4:**
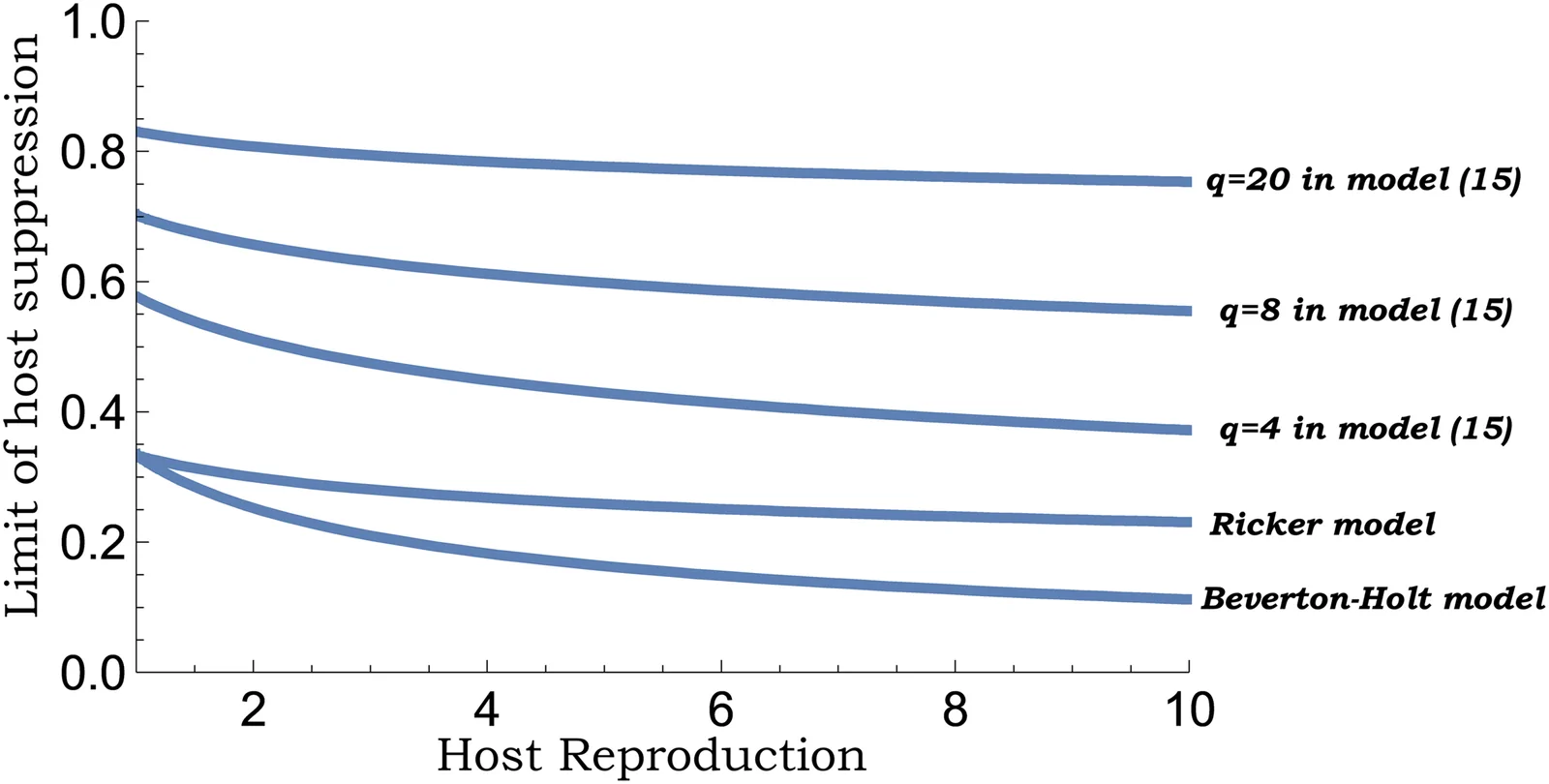
The limit of host suppression as quantified by the ratio of the lowest possible host density with and without parasitoid, conditioned on stable species coexistence. The limit is obtained from (45) as a function of host reproduction *R* for different models of parasitoid-free host population dynamics. The different models include the Beverton-Holt and Ricker models (*q* = 1 and q→∞ in (5), respectively) and (15) for different values of *q*.

Intriguingly, when R≈1, for $f_{H}$ of the form (5), this limit is always one-third independent of the value of *q* (Fig 4), and hence true for both Beverton-Holt and Ricker models. However, differences emerge for larger values of *R*, where decay in the limit is much more gradual for the Ricker model as compared to the Beverton-Holt model. When $f_{H}$ takes the form (15) then this limit of host suppression is


$$\begin{array}{c} {\left(\frac{1}{1+2q}\right)}^{1/q} \end{array}$$ (48)

when R≈1 (see S1 Appendix C in S1 File for proof), and the limit increases to one as q→∞ (Fig 4).

### Inclusion of stabilizing factors

We next explore the stabilizing effects of host growth self-limitation in combination with other stabilizing factors, such as host refuge. Such refuges can arise from a variety of scenarios, such as spatial occlusion of some hosts preventing access to parasitoids [49], or some hosts having higher immune response to parasitism [50–53]. Towards that end we consider the escape response (33) resulting in the model


$$\begin{array}{cc} H_{t+1} & =RH_{t}f_{H}(H_{t})\left(\mu +(1-\mu )e^{-cP_{t}}\right) \end{array}$$ (49a)

$$\begin{array}{cc} P_{t+1} & =kRH_{t}f_{H}(H_{t})(1-\mu )(1-e^{-cP_{t}}), \end{array}$$ (49b)

with parasitoid establishment requiring


$$\begin{array}{c} -kK\frac{df_{P}(P_{t})}{dP_{t}}{|}_{P_{t}=0}>1\;⟹\;c>\frac{1}{(1-\mu )kK}. \end{array}$$ (50)

The stabilization region in terms of $S_{H}(H^{*})$ and $cP^{*}$ is shown to considerably expand as a function of increasing refuge fraction μ (Fig 5).

**Fig 5:**
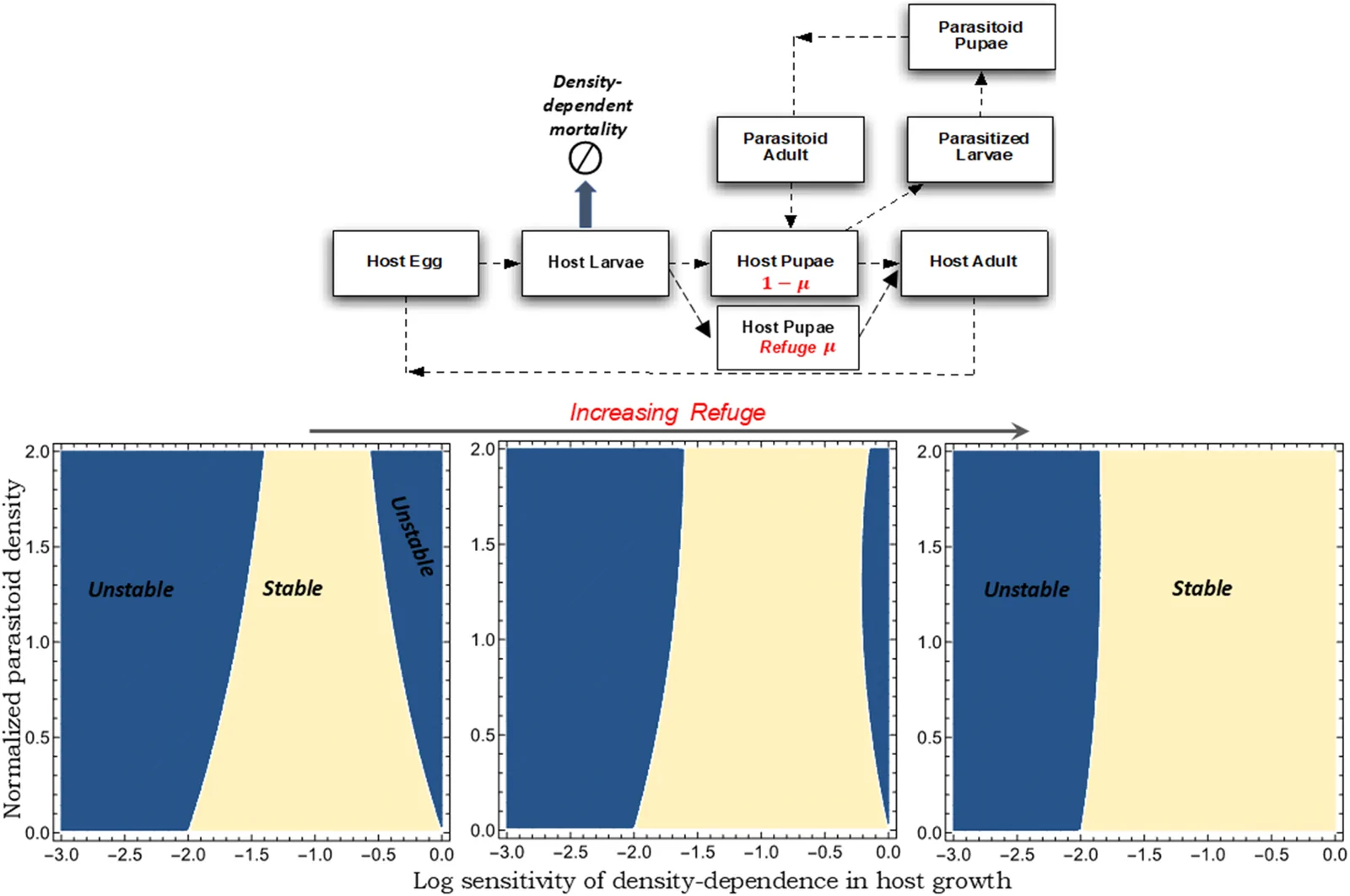
Schematic of the insect life cycles as in Fig 1, but showing a fraction μ of hosts protected from parasitism (i.e., in a refuge). The stability region as given by inequalities (23) for increasing values of μ. The left-most plot corresponds to μ=0 and is the same as in Fig 3. The middle and rightmost plots correspond to μ=0.15 and μ=0.5, respectively.

While there are subtle changes to the left instability-stability boundary, the right-hand boundary shifts further right and disappears for sufficiently large refuge μ. The disappearance can be understood from the fact that when μ>0.5, then inequality (23a) always holds as $1-f_{P}(P^{*})+S_{P}(P^{*})>0$. The stability regions in terms of μ and attack rate *c* are illustrated in Fig 6.

**Fig 6:**
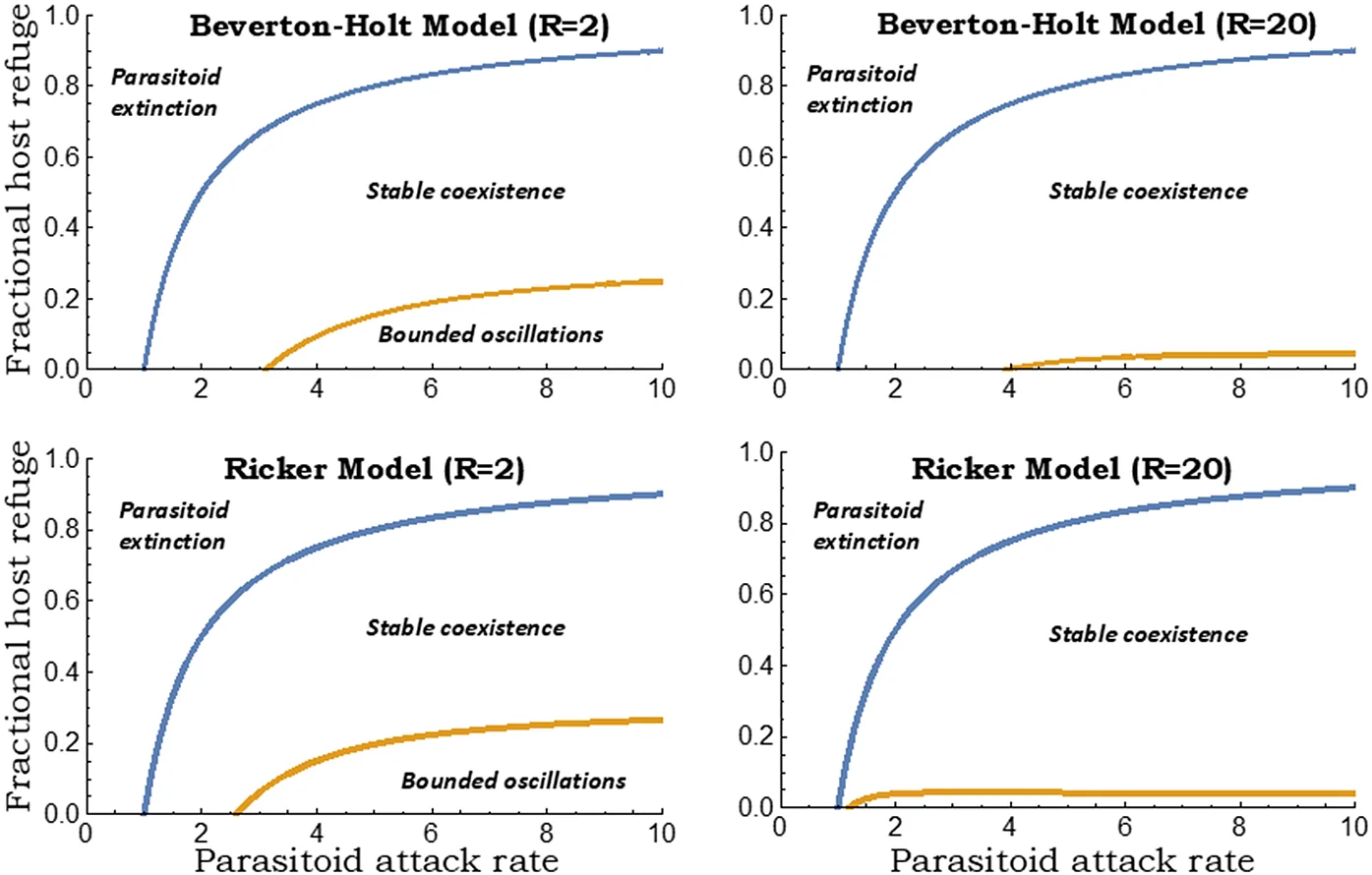
The region of stable host-parasitoid population dynamic for model (49) in terms of the fractional host refuge μ and parasitoid attack rate *c.* The regions are plotted for *R* = 2 (left) and *R* = 20 (right) where $f_{H}$ takes the form of the Beverton-Holt model (top) and the Ricker model (bottom). The blue line corresponds to (50), and the region above leads to parasitoid extinction. Stable consumer-resource coexistence occurs in the region in between the blue and orange lines, with the region below the orange line leading to bounded oscillations in population densities. Other parameters are taken as *K* = *k* = 1. Note that when the host density is sufficiently suppressed for high attack rates, the stability regions for Beverton-Holt and Ricker models become similar for a given *R* due to the weakening effect of self-limitation in host growth.

Note that the region allowing stable coexistence expands for the Beverton-Holt model with increasing *R*. In contrast, this region contracts with increasing *R* for μ≈0 for the Ricker model but expands for higher values of μ. The observed large regions of stable coexistence in the presence of fractional refuge and different forms of host growth self-limitation are consistent with experimental studies implicating refuges in the stability of host-parasitoid interactions [54–58]. We refer the reader to the preprint version of this manuscript [59] for stability regions in the case of parasitoid interference, where the escape response takes the form (30). As in the case of host refuge, we also see an expansion in the stability region with increasing values of *m* (see Fig 6 in [59]).

### Model with handling time

Standard discrete-time frameworks typically assume continuous search via a Type I functional response [5–11]. Models incorporating finite handling time through Holling Type II responses demonstrate that handling constraints reduce regulatory control and destabilize population dynamics at high host densities [7,24,60–66]. However, these handling time effects have largely been evaluated in systems lacking explicit host self-limitation ($f_{H}(H_{t})=1$). We next extend the model (18) to incorporate a finite parasitoid handling time *h*. This introduces a Type II functional response to the model, allowing us to examine how handling time affects parasitoid-driven host suppression. The resulting model with Type II functional response is:


$$\begin{array}{cc} H_{t+1} & =RH_{t}f_{H}(H_{t})f_{P}(H_{t},P_{t}), \end{array}$$ (51a)

$$\begin{array}{cc} P_{t+1} & =kRH_{t}f_{H}(H_{t})\left(1-f_{P}(H_{t},P_{t})\right), \end{array}$$ (51b)

where


$$\begin{array}{c} f_{P}(H_{t},P_{t})=\exp\left(-\frac{cP_{t}T}{1+chRH_{t}f_{H}(H_{t})}\right). \end{array}$$ (52)

Here, *h* represents the parasitoid handling time per host (in units of time) and *T* is the total duration of the host vulnerable period. Throughout our analysis, we set *T* = 1 without loss of generality, so *h* can be directly interpreted as the handling time normalized to the host vulnerable period (e.g., *h* = 0.1 corresponds to 10% of *T*). *K* is the host carrying capacity. The parameters *c* and *k* remain as defined in model (1). In the limit h→0, the escape response reduces to a Type I functional response while preserving intrinsic host self-limitation (18).

At the parasitoid-free equilibrium $\left(H_{t},P_{t})=(K,0\right)$, the host population is at its carrying capacity *K*. Evaluating the conditions for parasitoid establishment (S1 Appendix D in S1 File) yields the minimum attack rate required for the parasitoid to grow from low densities, called the establishment threshold:


$$\begin{array}{c} c>\frac{1}{K(kT-h)}. \end{array}$$ (53)

The influence of handling time on the population dynamics is shown in Fig 7 for the Beverton–Holt (6) and Ricker (8) models. For the Beverton–Holt model (Fig 7A), attack rates below the establishment threshold (53) result in parasitoid extinction, with the host population converging to its parasitoid-free density *K* (yellow region). Attack rates above the establishment threshold yield stable coexistence (blue region). As the attack rate increases further, stability is lost, resulting in bounded oscillations (orange region). At very high attack rates (transition from the orange to the yellow region), the parasitoids overexploit the hosts and go extinct, allowing the host population to return to its carrying capacity. These distinct dynamical regimes and their boundaries were determined numerically by evaluating time series and solving the stability criteria (S1 Appendix E in S1 File).

**Fig 7:**
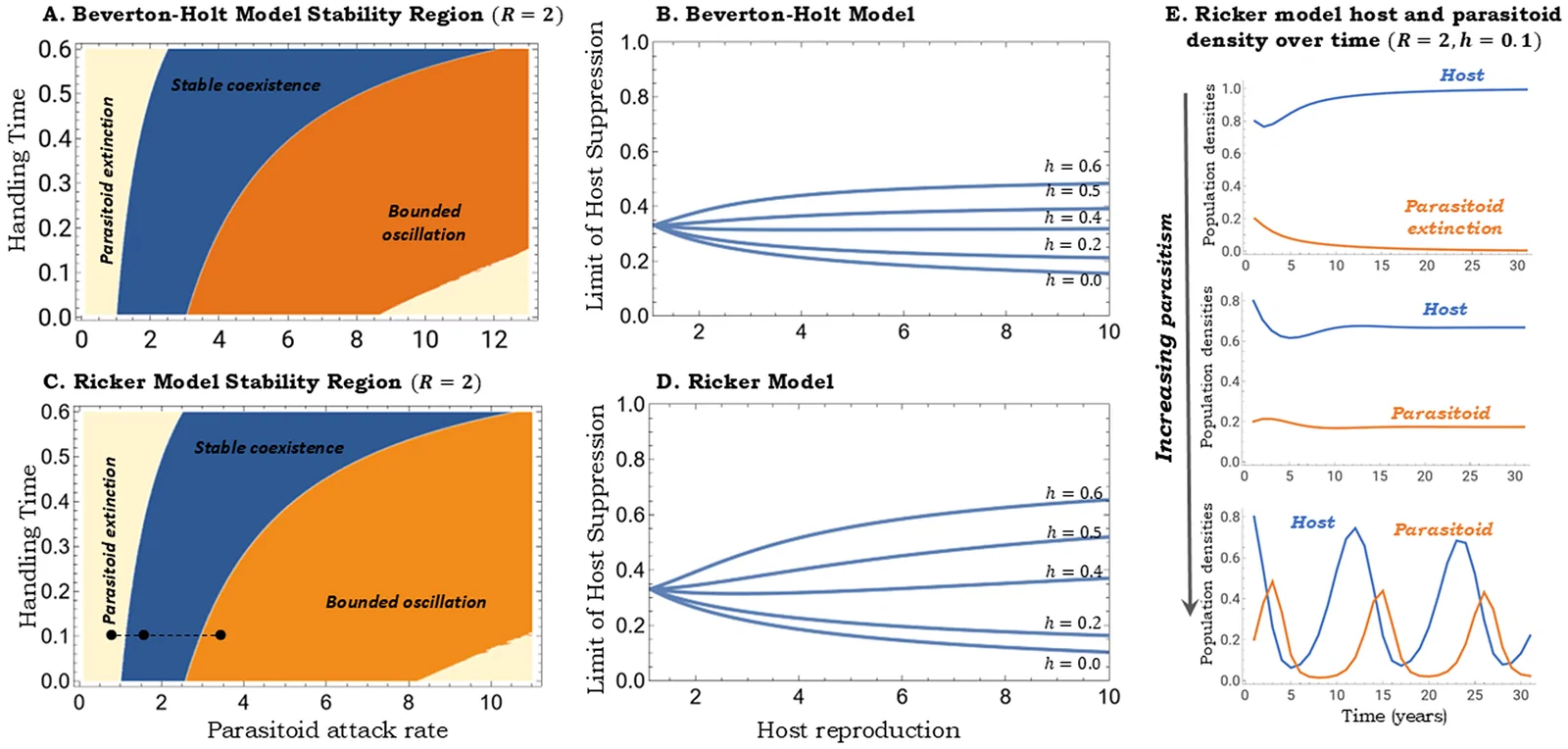
Influence of parasitoid handling time on host-parasitoid dynamics for model (51). (A) Stability diagram for the Beverton-Holt host growth model across parasitoid attack rate (*c*) and handling time (*h*). Attack rates below the establishment threshold (53) cause parasitoid extinction (yellow region), while higher attack rates enable stable species coexistence (blue region). At sufficiently high attack rates, the coexistence equilibrium loses stability, leading to bounded oscillations in population densities (orange region). Further increasing the attack rate (transition from the orange to the yellow region) results in unbounded population oscillations that ultimately lead to parasitoid extinction. (B) Host suppression limit ($H^{*}/K$) for the Beverton-Holt model as a function of host growth rate *R* for varying handling times *h*. When R≈1, $H^{*}/K=1/3$ regardless of handling time. At low *h*, host suppression limit decreases with increasing *R*, whereas at higher *h*, it increases with *R*. For a fixed *R*, the suppression limit increases with *h*. (C) Stability diagram for model (51) with the Ricker model for host growth, as a function of parasitoid attack rate *c* and handling time *h*. (D) Host suppression limit ($H^{*}/K$) for model (51), with the Ricker model, as a function of *R* for varying handling times *h*. (E) Representative population time series for the Ricker model (*R* = 2, *h* = 0.1) corresponding to marked points in C: parasitoid extinction at *c* = 1, stable coexistence at *c* = 1.5, and bounded oscillations at *c* = 3.5. The stability regions in panels A and C were obtained from local stability and time series analysis (S1 Appendix E in S1 File). Parameter values: *K* = 1, *k* = 1, *T* = 1.

The host suppression limit for the Beverton-Holt model is shown in Fig 7B. Recall that we defined the host suppression limit in Fig 4 as the host density (normalized to carrying capacity) when the population dynamics transition from a stable to unstable regime for sufficiently high parasitoid attack rates (the switch from the blue stability to the orange oscillatory region in Fig 7A). At low handling times *h*, host suppression limit decreases with increasing *R*, whereas at higher *h*, host suppression limit increases with *R*. For a fixed *R*, the host suppression limit increases with *h* (Fig 7B).

An interesting simplification occurs in the host suppression limit when R≈1. In this regime, $H^{*}/K=1/3$, regardless of the specific host growth function $f_{H}$ in (5) (S1 Appendix F in S1 File). So, both the Beverton–Holt and Ricker models reach the same suppression limit as found earlier for *h* = 0 (see Fig 4). This result extends beyond these two models. For any host self-limitation function $f_{H}(H_{t})$ satisfying (15), the suppression limit at R≈1 is


$$\begin{array}{c} \frac{H^{*}}{K}={\left(\frac{1}{1+2q}\right)}^{1/q}, \end{array}$$ (54)

which is identical to the *h* = 0 result in (48) (S1 Appendix G in S1 File). The Ricker model follows the same overall pattern as the Beverton-Holt model (Fig 7C). Our results also show higher sensitivity of the host suppression limit to increasing *h* at high host growth rates (Fig 7D) for the Ricker model compared to Beverton-Holt.

Fig 7E illustrates the population dynamics via time series for parameter regimes indicated by the black points in Fig 7C for the Ricker model. When the attack rate is low (*c* = 1), parasitoids cannot establish, allowing the host population to return to carrying capacity. Increasing the attack rate to *c* = 1.5 results in stable host-parasitoid coexistence. At the highest attack rate shown (*c* = 3.5), however, stronger parasitoid pressure destabilizes coexistence, leading to bounded oscillations in both populations.

Overall, a Type II functional response expands the range of parasitoid attack rates that support stable coexistence compared with the Type I case. Therefore, handling time increases the host suppression limit, and this effect is more pronounced at higher host growth rates. However, this increase remains small for biologically reasonable handling times. Thus, handling time affects the stability of host-parasitoid coexistence but has a comparatively minor effect on the limit of host suppression.

## Conclusion

In this contribution, we have systematically analyzed the population dynamics of host-parasitoid interactions with self-limitation in host growth. Models of the form (4) have previously been analyzed in the literature. For example [29], investigates the dynamical properties of a model similar to (4), where parasitism occurs after host self-limitation, but the analysis was restricted to $f_{H}$ given by the Beverton-Holt and Ricker models. Similarly [24,34], analyzed models with Beverton-Holt host population dynamics, with parasitoid attacks occurring either before, concurrently, or after host self-limitation. The key contributions of this work can be summarized as

- Analysis of the model (4) with the general forms of $f_{H}$ given by (5) and (15). In these forms, the parameter *q* can be modulated to capture different $f_{H}$ shapes (Fig 2) and ecological mechanisms, such as frequency-dependent predation.
- The derivation of necessary and sufficient conditions for the stable coexistence of both species as given by (23). The stable coexistence region as given by (23) is mapped out in Fig 3 in a way that is not linked to a specific $f_{H}$.
- Formulation of the limits of host suppression beyond which the population dynamics is destabilized. This limit is determined for different functions $f_{H}$ and *R* (Fig 4) and clearly shows a strong dependence on the shape of $f_{H}$.
- Studying the impact of including additional stabilizing mechanisms, such as host refuge (a fraction of the host population is protected from parasitism), and mapping out the shifts in the coexistence region with increasing refuge (Figs 5 and 6).
- Extending the above analysis to models of the form (51)-(52) that consider Type II functional responses, and determining the limits of host suppression for different parasitoid handling times (Fig 7).

Our results show that when parasitoids parasitize hosts randomly as per a Type I functional response, the stabilizing effect of host growth self-limitation is seen in Fig 3, with stability ensuing for a range of values of $S_{H}$ (the strength of density-dependence in host growth). When this strength of density-dependence is sufficiently weak such that $S_{H}>-1$ (as in the Beverton-Holt model or if the host density is operating sufficiently below its carrying capacity), then stability can be tied to a single inequality (42), with the stability region expanding with more negative values of $S_{H}$. As the equilibrium host density $H^{*}\to 0$ then from (13) and (16) the strength $S_{H}(H^{*})\to 0$, and (42) is violated for the Nicholson-Bailey escape response as in that case


$$\begin{array}{c} 1-f_{P}(P^{*})+S_{P}(P^{*})<0. \end{array}$$ (55)

This instability for sufficiently large parasitism sets up a limit of host density suppression that has fundamental consequences for biological control measures that use natural enemies such as parasitoids for reducing the density of arthropod pests [44,67–73]. A key result of this paper is that for a broad class of intrinsic host population dynamic models given by (5), we show that this limit is one-third of the host’s parasitoid-free density, independent of *q* for slow-growing hosts (R≈1) (Fig 4). However, for fast-growing hosts, this will depend on the specific value of *q*. For example, for *R* = 10 this limit is ≈10% for the Beverton-Holt model (*q* = 1) and ≈25% for the Ricker model (q→∞). In contrast, when density-dependent host growth takes the form as in (15) then this limit always depends on *q*, and increases with increasing *q* (Fig 4). *These results argue that the form of density-dependent host growth is important for the success of any biological control program and must be taken into account*.

The emergence of the one-third limit of host suppression for R≈1 and $f_{H}$ given by (5) is a mathematical consequence of the stability analysis. It represents the host density (normalized by its parasitoid-free density) at the stability-instability boundary, where further enhancing the parasitoid attack rate destabilizes the stable coexistence of both populations (Fig 3). This point is illustrated in the population density simulations in Fig 3, where a critical parasitoid attack rate is first needed for parasitoid establishment, coexistence is maintained over a range of attack rates, and a high attack rate destabilizes coexistence, resulting in population oscillations. Further increasing the attack rate will amplify these oscillations, making the parasitoid more prone to extinction in the presence of environmental stochasticity. It is interesting to contrast this one-third limit of host suppression with scenarios in which the timing of parasitism is altered relative to the arthropod host’s life cycle stage. Intriguingly, as shown for the Beverton-Holt model, this one-third limit is robust to parasitism timing as long as R≈1 [34]. However, for large values of *R*, the timing of parasitism affects the host suppression limit. For example, when *R* = 10, the limit of host suppression is 10% for the Beverton-Holt model (Fig 4; parasitism occurs after host self-limitation), but this limit increases to 30% and 55% when parasitism acts concurrently or before the host self-limitation stage, respectively [34]. These results reinforce the points that the timing of parasitism and host reproduction, in conjunction with the functional form of $f_{H}$, play a critical role in determining the success of parasitoid-driven biological control.

We further extended these results to account for additional stabilizing factors, such as host refuge and parasitoid interference, that alter the model’s escape response. The expansion of the coexistence stability region, achieved by coupling these factors with density-dependent host growth, is shown in Figs 3-5. When these factors are present such that the inequality (43) holds, stable parasitoid presence is ensured at high parasitism levels, leading to better suppression of host density.

The above analysis has been restricted to a Type I functional response for the parasitoid, and a natural question that arises is how robust are the limits of host suppression in Fig 4 to parasitoid handling times that result in Type II functional responses. Towards that end, we extended the analysis to models of the form (51)-(52) that combine self-limitation in host growth with a Type II functional response. Our analysis reveals that with handling time *h* > 0, the range of parasitoid attack rates allowing for stable coexistence expands compared to *h* = 0 (i.e., Type I functional response); still, the limit of host suppression increases with increasing *h* (Fig 7). Thus, while parasitoid handling times can have a detrimental impact on the biological control of host populations, the upward shift in the host suppression limits is small even for reasonably large values of the handling time, as can be seen by comparing the lines for *h* = 0 and *h* = 0.2 (handling time is 20% of the total host vulnerable period) in Figs 7B and D.

While this work has focused on the question of stable coexistence, future work will consider incorporating intraspecific competition between parasitoids [74], spatial dynamics connecting metapopulations, and exploring how spatial effects alter the fundamental limits of biological control [75–78]. It will also be important to explicitly consider parasitoid extinction in stochastic formulations of these models with both environmental fluctuations in parameters, such as the host reproduction *R*, and demographic stochasticity. Given the nonlinearities present in these models, the analysis will depend on both stochastic simulations and several closure schemes developed for analyzing population dynamic models [79–85]. Finally, it will be important to expand this analysis to Type II and Type III functional responses, which would require semi-discrete formulations that mechanistically capture the continuous changes in population densities during the host’s vulnerable stage [24,86,87].

## Supporting information

S1 File Appendix A–G containing detailed derivations of theoretical results. (PDF)
