# Supplementary material for: The form of density-dependence in host growth impacts parasitoid-driven host suppression
Source: PLoS One. 2026 Nov 5;21(11):e0358117. doi: 10.1371/journal.pone.0358117 (PMC13739278; doi:10.1371/journal.pone.0358117)
Supplement: Supplementary file 1 [file pone.0358117.s001.pdf]

Supplementary Information: *The form of density-dependence in host growth impacts parasitoid-driven host suppression*

Abhyudai Singh [1,2,3,4], Poulami Chatterjee [1]

1 Department of Electrical and Computer Engineering, University of Delaware, Newark, DE, United States
2 Department of Biomedical Engineering, University of Delaware, Newark, DE, United States
3 Department of Mathematical Sciences, University of Delaware, Newark, DE, United States
4 Center for Bioinformatics and Computational Biology, University of Delaware, Newark, DE, United States

* absingh@udel.edu

Appendix A

The consumer-resource model is given by

$$H_{t+1} = RH_t f_H(H_t) f_P(P_t), \tag{S1a}$$
$$P_{t+1} = kRH_t f_H(H_t)\left(1 - f_P(P_t)\right). \tag{S1b}$$

The stability of an equilibrium is determined by the Jacobian matrix A, obtained by linearizing the nonlinear terms on the right-hand side of (S1) around that equilibrium. We first consider the boundary equilibrium $(H_t, P_t) = (K, 0)$, which represents parasitoid extinction and host persistence at the carrying capacity K. The corresponding Jacobian matrix is

$$A = \begin{bmatrix} a_{00} & a_{01} \\ a_{10} & a_{11} \end{bmatrix} \tag{S2}$$

where the entries of the matrix are given by

$$a_{00} = \frac{\partial RH_t f_H(H_t) f_P(P_t)}{\partial H_t}\Big|_{H_t=K, P_t=0} = 1 + S_H(K), \tag{S3a}$$
$$a_{01} = \frac{\partial RH_t f_H(H_t) f_P(P_t)}{\partial P_t}\Big|_{H_t=K, P_t=0} = K f'_P(0) \tag{S3b}$$
$$a_{10} = k\frac{\partial RH_t f_H(H_t)\left(1 - f_P(P_t)\right)}{\partial H_t}\Big|_{H_t=K, P_t=0} = 0, \tag{S3c}$$
$$a_{11} = k\frac{\partial RH_t f_H(H_t)\left(1 - f_P(P_t)\right)}{\partial P_t}\Big|_{H_t=K, P_t=0} = -ka_{01}, \tag{S3d}$$

and

$$f'_P(0) := \frac{df_P(P_t)}{dP_t}\Big|_{P_t=0}. \tag{S4}$$

Because $a_{10} = 0$, the Jacobian matrix A is upper triangular. Consequently, its eigenvalues are simply given by its diagonal entries, a_{00} and a_{11}. This direct analysis shows that the fixed point $(H^*, P^*) = (K, 0)$ is stable if and only if,

$$-2 < S_H(K) < 0, \quad -1 < kKf'_P(0) \leq 0. \tag{S5}$$

The second inequality arises from the fact that when $H_t = K$,

$$\lim_{P_t \to 0} \frac{P_{t+1}}{P_t} = -kKf_P'(0), \tag{S6}$$

and $-kKf_P'(0) < 1$ leads to parasitoid extinction starting from small parasitoid densities. This result leads to a condition for parasitoid establishment:

$$-kKf_P'(0) > 1 \tag{S7}$$

is a necessary and sufficient condition for parasitoids to grow from small numbers.

Appendix B

To determine the local stability of the coexistence fixed point, we linearize the system around $(H_t, P_t) = (H^*, P^*)$. The corresponding Jacobian matrix is given by,

$$a_{00} = 1 + S_H(H^*), \quad a_{01} = \frac{H^*}{P^*} S_P(P^*), \quad a_{10} = k(1 + S_H(H^*)) \left(\frac{1}{f_P(P^*)} - 1 \right), \quad a_{11} = -ka_{01}. \tag{S8}$$

Using the equilibrium relationship $RH^* f_H(H^*) f_P(P^*) = H^*$, the identity $a_{11} = -ka_{01}$ follows. The trace and determinant of the Jacobian are,

$$\mathrm{tr}(A) = a_{00} + a_{11}, \quad \det(A) = a_{00}a_{11} - a_{01}a_{10}. \tag{S9}$$

For a two-dimensional discrete-time system, the Jury stability criteria require that both eigenvalues of A lie inside the unit circle. Biologically, these conditions determine whether small perturbations away from the equilibrium decay over time (stable coexistence) or amplify (loss of stability and population oscillations). These conditions can be expressed in terms of the trace and determinant as,

$$1 - \mathrm{tr}(A) + \det(A) > 0, \quad 1 + \mathrm{tr}(A) + \det(A) > 0, \quad 1 - \det(A) > 0. \tag{S10}$$

Substituting the expressions for the Jacobian entries into these three inequalities and simplifying gives the following necessary and sufficient conditions for stability of the coexistence fixed point:

$$1 - f_P(P^*) + S_P(P^*) > -S_H(H^*) S_P(P^*), \tag{S11a}$$

$$S_H(H^*)(-1 + f_P(P^*) - S_P(P^*)) - (1 - f_P(P^*))\, S_P(P^*) > 0, \tag{S11b}$$

$$S_H(H^*)(1 - f_P(P^*) - S_P(P^*)) + 2\,(1 - f_P(P^*)) - S_P(P^*)\,(1 + f_P(P^*)) > 0, \tag{S11c}$$

where S_H and S_P are the dimensionless log sensitivities of the functions f_H and f_P, respectively,

$$S_H(H^*) := \frac{H^*}{f_H(H^*)} \frac{df_H(H_t)}{dH_t}\Big|_{H_t = H^*} < 0, \quad S_P(P^*) := \frac{P^*}{f_P(P^*)} \frac{df_P(P_t)}{dP_t}\Big|_{P_t = P^*} < 0. \tag{S12}$$

We investigate these inequalities in further detail for different scenarios, highlighting new results and connecting them with known results.

Appendix C

The previous sections established general stability criteria. Here we apply these criteria to specific density-dependent host growth functions to determine how host suppression depends on the functional form

of $f_H(H)$. We consider the escape response $f(P_t) = e^{-cP_t}$ as in the Nicholson-Bailey model where at equilibrium

$$f_P(P^*) = \frac{1}{Rf_H(H^*)} \implies cP^* = \log Rf_H(H^*). \tag{S13}$$

The population dynamics is destabilized for sufficiently large parasitoid attack rates c, and at the stability-instability boundary

$$\frac{1 - e^{-cP^*} - cP^*}{cP^*} = -S_H(H^*). \tag{S14}$$

From (S13), in the limit $R \to 1$, $P^* \to 0$, and the left-hand-side of (S14) reduces to

$$\lim_{P^* \to 0} \frac{1 - e^{-cP^*} - cP^*}{cP^*} = -\frac{cP^*}{2} = -\frac{\log Rf_H(H^*)}{2} = -\frac{\log R + \log f_H(H^*)}{2}. \tag{S15}$$

For the density-dependent growth function defined in (5) in the main text, when $R \approx 1$

$$\log f_H(H^*) = -q \log\left(1 + \frac{\left(R^{\frac{1}{q}} - 1\right) H^*}{K}\right) \approx -q \frac{\left(R^{\frac{1}{q}} - 1\right) H^*}{K} \approx -\frac{(R-1)\, H^*}{K}. \tag{S16}$$

Note here we have used the fact that when $R \approx 1$, then $R^{\frac{1}{q}} - 1 \approx (R-1)/q$. Similarly, using (13) in the main text, we can approximate $S_H(H^*)$ when $R \approx 1$ as

$$S_H(H^*) = -q\left(1 - \frac{1}{1 + \left(R^{\frac{1}{q}} - 1\right)\frac{H^*}{K}}\right) \approx -q\left(R^{\frac{1}{q}} - 1\right)\frac{H^*}{K} \approx -(R-1)\frac{H^*}{K}. \tag{S17}$$

Substituting (S15)-(S17) back in (S14), and using $\log R \approx R - 1$ yields

$$\frac{\log R + \log f_H(H^*)}{2} = (R-1)\frac{H^*}{K} \implies (R-1) - \frac{(R-1)\, H^*}{K} = 2(R-1)\frac{H^*}{K} \implies \frac{H^*}{K} = \frac{1}{3}. \tag{S18}$$

Thus, independent of the value of q, for $R \approx 1$, at the stability-instability boundary the host density is one-third of its parasitoid-free density.
Repeating a similar analysis for $R \approx 1$ but with f_H of the form (15) in the main text yields

$$\log f_H(H^*) = -\log\left(1 + (R-1)\left(\frac{H^*}{K}\right)^q\right) \approx -(R-1)\left(\frac{H^*}{K}\right)^q, \tag{S19}$$

and from (16) in the main text we get,

$$S_H(H_t) = -q\left(1 - \frac{1}{1 + (R-1)\left(\frac{H_t}{K}\right)^q}\right) \implies S_H(K) = -q\left(1 - \frac{1}{R}\right). \tag{S20}$$

which gives the host suppression limit as:

$$\frac{\log R + \log f_H(H^*)}{2} = q(R-1)\left(\frac{H^*}{K}\right)^q \tag{S21a}$$

$$\implies (R-1) - (R-1)\left(\frac{H^*}{K}\right)^q = 2q(R-1)\left(\frac{H^*}{K}\right)^q \tag{S21b}$$

$$\implies \frac{H^*}{K} = \left(\frac{1}{1+2q}\right)^{1/q}. \tag{S21c}$$

Appendix D

This appendix extends the parasitoid establishment condition in Appendix A to the Type II functional response model given by Eq. (51)–(52) in the main text. The escape response is

$$f_P(H_t, P_t) = \exp\left(-\frac{cP_tT}{1 + chRH_tf_H(H_t)}\right). \tag{S22}$$

When $h = 0$, this reduces to the Type I Nicholson-Bailey escape response used in (28) of the main text. For $h > 0$, the denominator accounts for handling time, introducing a Type II functional response with a density-dependent modified attack rate. At the parasitoid-free equilibrium, $(H^*, P^*) = (K, 0)$, the host satisfies $Rf_H(K) = 1$, and the escape response satisfies $f_P(K, 0) = 1$. Following the linearization approach in Appendix B, the Jacobian matrix evaluated at the boundary equilibrium simplifies to an upper-triangular structure:

$$A = \begin{bmatrix} 1 + S_H(K) & -\frac{cKT}{1+chK} \\ 0 & \frac{kcTK}{1+chK} \end{bmatrix}, \quad \text{where } S_H(K) = \frac{K}{f_H(K)} \left.\frac{df_H(H_t)}{dH_t}\right|_{H_t=K} \tag{S23}$$

Hence, its eigenvalues are simply the diagonal entries, $1 + S_H(K)$, and $\frac{kcTK}{1+chK}$. The first eigenvalue $1 + S_H(K)$ is stable under the condition in (S12), where $S_H(K) < 0$. Therefore, parasitoid establishment is determined by the second eigenvalue, $\frac{kcTK}{1+chK}$. Expressing this growth factor via the derivative of the escape response $f'_P(K, 0) = -\frac{cT}{1+chK}$ confirms exact consistency with the condition as in (S6):

$$\lim_{P_t \to 0} \frac{P_{t+1}}{P_t} = -kKf'_P(K, 0) = a_{11} = \frac{kcTK}{1 + chK} \tag{S24}$$

Consequently, parasitoid establishment from low initial densities requires:

$$\frac{kcTK}{1 + chK} > 1 \implies kcTK > 1 + chK \implies cK(kT - h) > 1 \tag{S25}$$

This gives the necessary and sufficient condition for establishment of parasitoids from arbitrarily small initial densities:

$$c > \frac{1}{K(kT - h)}, \tag{S26}$$

Setting h=0 in (S26) recovers the establishment threshold derived for the parasitoid escape response (44) in the main text.

Appendix E

This appendix derives the coexistence equilibrium and its local stability for the Beverton–Holt host model with a Type II functional response using the Jury conditions from Appendix B.

$$H_{t+1} = RH_tf_H(H_t)f_P(H_t, P_t), \tag{S27a}$$

$$P_{t+1} = kRH_tf_H(H_t)\left(1 - f_P(H_t, P_t)\right), \tag{S27b}$$

where

$$f_H(H_t) = \frac{1}{1 + \frac{(R-1)H_t}{K}}, \qquad f_P(H_t, P_t) = \exp\left(-\frac{cP_tT}{1 + chRH_tf_H(H_t)}\right). \tag{S28}$$

Setting $h = 0$ recovers the Nicholson-Bailey (Type I) escape response given by (28) in the main text, whereas $h > 0$ corresponds to the Type II functional response analyzed in this section. We first derive the coexistence equilibrium $(H_t, P_t) = (H^*, P^*)$,

$$P^* = \left(\frac{1 + chRH^* f_H(H^*)}{cT}\right) \ln\left(\frac{RH^* f_H(H^*)}{H^*}\right). \tag{S29}$$

Substitution into the host equilibrium condition produces the implicit equation

$$RH^* f_H(H^*)\left[1 - \frac{H^*}{RH^* f_H(H^*)}\right] - \left(\frac{1 + chRH^* f_H(H^*)}{cT}\right) \ln\left(\frac{RH^* f_H(H^*)}{H^*}\right) = 0. \tag{S30}$$

A biologically meaningful coexistence equilibrium requires $H^* > 0$, $P^* > 0$, and $H^* < K$. For the Beverton-Holt model, the coexistence equilibrium is obtained from the implicit equation above. The left-hand side is monotonic in H^*, providing at most one positive solution. Therefore, when the parasitoid establishment condition is satisfied, the coexistence equilibrium is unique. Below the establishment threshold in (S26), parasitoids cannot invade from an arbitrarily low density for host reproduction, and the system converges to the parasitoid free equilibrium. For the Ricker model, where host density dependence follows $f_H(H_t) = R^{1-H_t/K}$, the coexistence equilibrium is

$$P^* = \frac{1 + chH^* R^{1-\frac{H^*}{K}}}{cT} \ln\left(R^{1-\frac{H^*}{K}}\right). \tag{S31}$$

Substituting into the parasitoid equilibrium equation gives the host equilibrium condition

$$\frac{R^{-H^*/K}(chH^* R + R^{H^*/K}) \ln\left(R^{1-\frac{H^*}{K}}\right)}{cT} = H^*\left(-1 + R^{1-\frac{H^*}{K}}\right). \tag{S32}$$

At the coexistence equilibrium (H^*, P^*), the host equation satisfies $H^* = RH^* f_H(H^*) f_P(H^*, P^*)$. Dividing by H^* and isolating the exponential term yields,

$$\exp\left(\frac{cP^* T}{1 + chRH^* f_H(H^*)}\right) = \frac{H^*}{RH^* f_H(H^*)}. \tag{S33}$$

Taking the negative natural logarithm of both sides defines the modified attack rate as

$$\left(\frac{cP_t T}{1 + chRH_t f_H(H_t)}\right) = \ln\left(\frac{RH^* f_H(H^*)}{H^*}\right) \tag{S34}$$

For the fixed point $(H_t, P_t) = (H^*, P^*)$ the entries for the Jacobian matrix are,

$$a_{00} = 1 + S_H(H^*) + \bar{S}_H(H^*, P^*), \quad a_{01} = \frac{H^*}{P^*}\bar{S}_P(H^*, P^*), \tag{S35a}$$

$$a_{10} = k\left[(1 + S_H(H^*))\left(\frac{1}{f_P(H^*, P^*)} - 1\right) - \bar{S}_H(H^*, P^*)\right], \quad a_{11} = -ka_{01}, \tag{S35b}$$

where

$$S_H(H^*) = \frac{H^*}{f_H(H^*)} \frac{df_H(H_t)}{dH_t}\bigg|_{H_t = H^*} = -\frac{(R-1)H^*}{K + (R-1)H^*}, \tag{S36a}$$

$$\bar{S}_H(H^*, P^*) = \frac{H^*}{f_P(H^*, P^*)} \frac{\partial f_P(H_t, P_t)}{\partial H_t}\bigg|_{H_t = H^*,\, P_t = P^*} = \frac{chRH^* f_H(H^*)}{1 + chRH^* f_H(H^*)} \ln\left(\frac{RH^* f_H(H^*)}{H^*}\right), \tag{S36b}$$

$$\bar{S}_P(H^*, P^*) = \frac{P^*}{f_P(H^*, P^*)} \frac{\partial f_P(H_t, P_t)}{\partial P_t}\bigg|_{H_t = H^*,\, P_t = P^*} = -\frac{cP^* T}{1 + chRH^* f_H(H^*)}. \tag{S36c}$$

Here, S_H denotes the logarithmic sensitivity of the density-dependent host growth function $f_H(H)$, whereas $\bar{S}_H$ and $\bar{S}_P$ denote the logarithmic sensitivities of the two-variable parasitoid escape response $f_P(H,P)$ with respect to host and parasitoid densities, respectively. Using the relationship $a_{11} = -ka_{01}$, the determinant becomes

$$\det(A) = -a_{01}(ka_{00} + a_{10}) = -k\frac{H^*}{P^*}\frac{\bar{S}_P(H^*,P^*)\,(1+S_H(H^*))}{f_P(H^*,P^*)} \tag{S37}$$

The trace of the Jacobian is given by

$$\mathrm{tr}(A) = 1 + S_H(H^*) + \bar{S}_H(H^*,P^*) - k\frac{H^*}{P^*}\bar{S}_P(H^*,P^*). \tag{S38}$$

The local stability of the coexistence equilibrium is again determined by the Jury criteria introduced in Appendix B, using the trace and determinant of the Jacobian derived above. Handling time influences stability through the attack rate,

$$\frac{cP^*T}{1 + chRH^*f_H(H^*)}, \tag{S39}$$

which appears in both the trace and determinant. Increasing h increases the denominator of the escape response, reducing parasitoid efficiency and shifting the boundaries between stable and unstable coexistence as determined by the Jury conditions.

Appendix F

This appendix proves that near $R \approx 1$, the host suppression limit converges to $H^*/K = 1/3$ for the host density-dependent growth functions of the form in (5) in the main text. We evaluate the system at the stability boundary where $\det(A) = 1$. Substituting the log-sensitivities from (S36) into the determinant (S37) yields:

$$\det(A) = -k\frac{H^*}{P^*}\frac{\bar{S}_P(H^*,P^*)\,(1+S_H(H^*))}{f_P(H^*,P^*)} = \frac{\ln\left(Rf_H(H^*)\right)}{Rf_H(H^*) - 1}\cdot\frac{d}{dH_t}\left(RH_tf_H(H_t)\right)\Big|_{H_t=H^*}. \tag{S40}$$

Let $R = 1 + \epsilon$ and define the host suppression ratio as $x = H^*/K$. Expanding

$$(1+\epsilon)^{1/q} \approx 1 + \frac{\epsilon}{q} - \frac{q-1}{2q^2}\epsilon^2 \implies R^{1/q} - 1 \approx \frac{\epsilon}{q}. \tag{S41}$$

As $\epsilon \approx 0$, the density-dependent growth term becomes:

$$Rf_H(H^*) = (1+\epsilon)\left(1 + \frac{\epsilon x}{q}\right)^{-q} \approx (1+\epsilon)(1-\epsilon x) \approx 1 + \epsilon(1-x) + \ldots \tag{S42}$$

This provides the first-order local approximations for the logarithmic ratio:

$$\frac{\ln\left(Rf_H(H^*)\right)}{Rf_H(H^*) - 1} \approx 1 - \frac{1}{2}\epsilon(1-x). \tag{S43}$$

Similarly, evaluating the derivative of the regulated host function yields:

$$\frac{d}{dH_t}\left(RH_tf_H(H_t)\right)\Big|_{H_t=H^*} = Rf_H(H^*) - R\epsilon x\left(1 + \frac{\epsilon x}{q}\right)^{-q-1} \approx 1 + \epsilon(1-2x). \tag{S44}$$

We evaluate the boundary at which the stable coexistence equilibrium loses stability through oscillations. For a two-dimensional discrete-time system, this transition occurs when a pair of complex eigenvalues crosses the unit circle, which corresponds to the condition $\det(A) = 1$.

$$\det(A) \approx \left[1 - \frac{1}{2}\epsilon(1-x)\right][1 + \epsilon(1-2x)] = 1 + \epsilon\left[(1-2x) - \frac{1}{2}(1-x)\right] = 1. \tag{S45}$$

For this condition to hold as $\epsilon \approx 0$, the first-order coefficient of ϵ must vanish:

$$(1-2x) - \frac{1}{2}(1-x) = 0 \implies 1 - 3x = 0 \implies x = \frac{H^*}{K} = \frac{1}{3}. \tag{S46}$$

Near the limit $R \approx 1$, the first-order expansion of the stability boundary is independent of both the handling time h and the Hill coefficient q, yielding the host suppression level $H^*/K = 1/3$. The same asymptotic analysis holds for the Beverton-Holt growth function (the special case where $q = 1$) and the Ricker growth function $f_H(H_t) = R^{1-H/K}$ (the limiting case as $q \to \infty$). Substituting either density-dependent growth function into the determinant condition and applying the limit $R \approx 1$ yields the identical first-order expansions $1 + \epsilon(1-x)$ and $1 + \epsilon(1-2x)$. Therefore, for the host density-dependent growth function defined in model (5), the host suppression ratio at the stability boundary converges to $H^*/K = 1/3$.

Appendix G

We now extend the host-suppression limit derived in Appendix C for the general growth function $f_H(H_t)$ defined in (15) in the main text to the Type II response with handling time h (52) in the main text. At the internal equilibrium (H^*, P^*) for the system in (51) in the main text, the model satisfies $Rf_H(H^*)f_P(H^*, P^*) = 1$, which gives:

$$f_P(H^*, P^*) = \frac{1}{Rf_H(H^*)}. \tag{S47}$$

Taking the natural logarithm of both sides defines the modified attack rate as a parameter z^*:

$$z^* \equiv \frac{cP^*T}{1 + chRH^*f_H(H^*)} = \ln\left(Rf_H(H^*)\right) = \ln R + \ln f_H(H^*). \tag{S48}$$

The log sensitivity of the host density function $f_H(H_t)$ is defined as:

$$S_H(H^*) = \frac{H^*}{f_H(H^*)}\frac{\mathrm{d}f_H(H_t)}{\mathrm{d}H_t}\bigg|_{H_t=H^*}. \tag{S49}$$

At the stability-instability boundary, the general characteristic equation reduces (in the limit $R \approx 1$ where $P^* \to 0$ and $z^* \to 0$) to:

$$\frac{1 - e^{-z^*} - z^*}{z^*} = -S_H(H^*). \tag{S50}$$

As $R \approx 1$, we have $z^* \to 0$, and the stability condition (S50) becomes

$$\frac{z^*}{2} = -S_H(H^*), \tag{S51}$$

using the first-order expansion

$$\frac{1 - e^{-z^*} - z^*}{z^*} \approx -\frac{z^*}{2}.$$

Substituting (S48) gives

$$\frac{\ln R + \ln f_H(H^*)}{2} = -S_H(H^*). \tag{S52}$$

Using the first-order approximations

$$\ln R \approx R - 1, \implies \ln f_H(H^*) \approx -(R-1)\left(\frac{H^*}{K}\right)^q, \implies S_H(H^*) \approx -q(R-1)\left(\frac{H^*}{K}\right)^q, \tag{S53}$$

yields

$$1 - \left(\frac{H^*}{K}\right)^q = (1+2q)\left(\frac{H^*}{K}\right)^q, \tag{S54}$$

and hence

$$\frac{H^*}{K} = \left(\frac{1}{1+2q}\right)^{1/q}. \tag{S55}$$

Equation (S55) corresponds to the analytical host suppression limit reported in (54) of the main text.
